# A weighted bootstrap approach to logistic regression modelling in identifying risk behaviours associated with sexual activity

**DOI:** 10.1080/17290376.2019.1636708

**Published:** 2019-07-02

**Authors:** Humphrey Brydon, Rénette Blignaut, Joachim Jacobs

**Affiliations:** a Department of Statistics and Population Studies, University of the Western Cape, Cape Town, South Africa; b HIV Unit, University of the Western Cape, Cape Town, South Africa

**Keywords:** HIV prevention, logistic regression, sample weighting, sexual risk behaviour, variable selection, weighted bootstrap

## Abstract

The latest population estimates released by Statistics South Africa indicate that 25.03% of all deaths in 2017 in South Africa were AIDS-related. Along with these results, it is also reported that 7.06% of the population were living with HIV, with the HIV-prevalence among youth (aged 15–24) at 4.64% for 2017 (STATSSA. (2018). Retrieved from Statistics South Africa: http://www.statssa.gov.za/publications/P0302/P03022017.pdf). The data used in the study contained information related to the risk-taking behaviours associated with the sexual activity of entering first-year students at the University of the Western Cape. In this study, a logistic regression modelling procedure was carried out on those students that were determined to be sexually active, therefore, in the modelling procedure significant risk behaviours of sexually active first-year students could be identified. Of the 14 variables included in the modelling procedure, six were found to be significantly associated with sexually active students. The significant variables included; the age and race of the student, whether the student had ever taken an HIV test, the importance of religion in influencing the sexual behaviour of the student, whether the student consumed alcohol and lastly whether the student smoked. This study further investigated the impact of introducing sample weighting, bootstrap sampling as well as variable selection methods into the logistic regression modelling procedure. It is shown that incorporating these techniques into the modelling procedure produces logistic regression models that are more accurate and have an increased predictive capability. The bootstrapping procedure is shown to produce logistic regression models that are more accurate than those produced without a bootstrap procedure. A comparison between 200, 500 and 1000 bootstrap samples is also incorporated into the modelling procedure with the models produced from 200 bootstrap samples shown to be just as accurate those produced from 500 or 1000 bootstrap samples. Of the five variable selection methods used, it is shown that the Newton–Raphson and Fisher methods are unreliable in producing logistic regression models. The forward, backward and stepwise variable selection methods are shown to produce very similar results.

## 1. Introduction

Identifying risk behaviours associated with sexual activity is of keen importance in helping curb the spread of Human Immunodeficiency Virus and Acquired Immunodeficiency Syndrome (HIV/AIDS). The aim of this study was to look at identifying the risk behaviours of sexually active entering first-year students at the University of the Western Cape (UWC) by utilising a logistic regression modelling procedure to identify these risk behaviours. By identifying the risk behaviours of these sexually active students, the results of this study could assist the HIV Unit at UWC with establishing a more targeted HIV prevention programme.

Logistic regression is a popular predictive modelling tool used in the cases where the event concerned is classified according to predefined classes. Even though logistic regression modelling is widely used, there remains a need for an effective method(s) to ensure that the model or models been produced are stable. As discussed by Austin and Tu, numerous logistic regression models can be produced from a single data set and this is further compounded with the introduction of variable selection methods (Austin & Tu, 2004a).

In this study sample weighting and bootstrapping procedures are introduced into the logistic modelling procedure along with variable selection methods to look at the effect that these procedures and methods have on the model or models produced.

In a study conducted by Luus, Neethling, and De Wet (2012) sample weighting techniques are been shown to produce reduced bias and Mean Square Errors (MSE) when used in logistic regression modelling procedures (Freedman & Berk, 2008; Luus et al., 2012). This reduction in the bias and MSE’s resulted in logistic regression models that were more accurate than those without a sample weighting technique.

Although there are numerous bootstrapping methods available, this study looked specifically at the bootstrap procedure of sampling with replacement (i.e. non-parametric bootstrap since the distribution family of the data is unknown). One of the advantages of incorporating a bootstrapping procedure in a modelling procedure is that no assumptions need to be made about the probability distribution of the original data set and the standard errors associated with the estimates of the bootstrap are all valid (Efron & Tibshirani, 1993).

In order to assess the fit of the logistic regression models produced to the data, a comparison is carried out on the goodness-of-fit statistics produced from each of the logistic regression models. Akaike’s Information Criterion (AIC) and the Bayesian (Schwartz) Information Criterion (BIC/SC) are used in order to assess the overall fit of the logistic regression models produced.

As with the bootstrapping procedure utilised in this study, when using the AIC and BIC/SC goodness-of-fit statistics the distribution of the data on which the logistic regression models are built does not need to be taken into consideration.

In this study the forward, backward, stepwise, Newton–Raphson and Fisher variable selection methods are used. Although the use of a variable selection method will not entirely omit the inclusion of predictor variables that are correlated, when used in conjunction with sample weighting and a bootstrapping procedure, they provide a model or models that are more accurate or fit the data better than if they had not been included (Austin & Tu, 2004a; Raftery, 1995).

## 2. Methods

### 2.1. Logistic regression

This study focussed on the binary logistic regression model which is the case where the dependent or outcome variable is specified to have only one of two possible outcomes (i.e. two classes). The logistic regression model used, assigned a probability to an observation based on its assigned outcome variable (i.e. the probability that the specific observation will have that specific outcome). The form of the log-likelihood function for multiple logistic regression can be seen in Equation (1) (Kleinbaum, 1994; Kutner, Nachtsheim, Neter, & Li, 2005):

(1) $$\mathrm{lo}g_{e}L(\beta )=\;\overset{n}{\underset{i=1}{\sum }}Y_{i}(X_{i}^{\prime }\beta )-\overset{n}{\underset{i=1}{\sum }}\mathrm{lo}g_{e}[1+\exp(X_{i}^{\prime }\beta )].$$

It is clear that the logistic regression model can involve many predictor variables ($X_{i})$ coupled with each of their coefficients β to predict the probability of a certain event or outcome. The coefficients (β) in Equation (1) are obtained by using the method of maximum likelihood estimation (MLE).

The MLE method uses an iterative algorithm that re-estimates the log likelihood until there appears to be minimal significant change in the residuals of the logit function (given in Equation (2)) (Kleinbaum, 1994; Kutner et al., 2005), i.e. MLE maximises the log likelihood function. The logit function is identical to that of the logistic regression model and is more commonly used in place of the logistic regression model as it is a more convenient equation to use (Kleinbaum, 1994; Kutner et al., 2005).

(2) $$\mathrm{logit}P(D|X_{1},\;\ldots ,\;X_{k})=ln_{e}\left[\frac{P(D|X_{1},\;\ldots ,\;X_{k})}{1-P(D|X_{1},\;\ldots ,\;X_{k})}\right].$$

Where $P(D|X_{1},\;\ldots ,\;X_{k})$ is the predicted probability that $Y_{i}$ will fall into either of the categories and k the number of variables under consideration. The shape of the distribution of the probability responses produced from the logistic regression model is important. What this shape shows is that there is a specific combination of predictor variables ($X_{i}$) and their coefficients (β), that have a significant effect on the probability response of the model but as this combination tends to either −∞ or +∞ this effect becomes minimal (Kleinbaum, 1994).

### 2.2. Ethics clearance

Ethical approval for the study was obtained from the University of the Western Cape Ethics Committee (nr 05/1/33). All students who participated in the study signed a consent letter prior to the completion of the anonymous questionnaire.

### 2.3. Bootstrapping and the data set characteristics

Non-parametric bootstrapping is one method of sampling from some original data set with replacement and creating what is called bootstrap samples. Ideally the number of bootstrap samples needs to be as large as possible; however this is not always possible as it can quite time consuming in assessing the bootstrap samples and the estimates obtained from these bootstrap samples.

In a study conducted by Luus et al. where 200 bootstrap samples were used, a good performance was reported of the estimators’ mean square error (MSE) and bias under bootstrap (Luus et al., 2012). Steyerberg et al. (2001) concluded that bootstrapping techniques provided more stable and nearly unbiased estimators of performance in logistic regression.

Since the bootstrap samples were constructed by resampling with replacement from a single data set it would be good to examine the characteristics of this data set as these same characteristics are expected to be carried over to the bootstrap samples in the bootstrap sampling procedure. The training and validation data sets were created by splitting the original (i.e. population) data set, which contained 1256 observations into 90% and 10% samples. The training data set contained 1130 observations (i.e. 90% of 1256) and the validation data set contained 126 observations (i.e. 10% of 1256) which was used for evaluating the logistic regressions models produced.

The data contained within the original data set was collected from entering first-year students at UWC during the orientation weeks in 2010. This data was collected by means of an anonymous questionnaire in which students voluntarily responded to 60 questions based on their attitude and behaviour towards certain situations. All students who completed the questionnaire were included in the study.

The questions contained within the questionnaire were either categorical or continuous. In order to ensure accuracy, a double capturing process was used. The capturing was done so in Microsoft Excel and the analysis was carried out in SAS® 9.4.

The gender and race profile of entering first-year students at UWC in 2010 can be seen in Table 1. A total of 6 observations were omitted from Table 1 due to missing values for either the race or gender variables. The frequency count for females within the training data set was quite large compared to that of the males within the training data set, females made up 63.5%, whereas males made up 36.5% of the training data set.

**Table 1.** Gender versus racial grouping in the training data set.

	Race				
	African/Black	Coloured	White	Indian/Asian	Total
Female	237 (count)	427	28	22	714
	33.19 (row %)	59.80	3.92	3.08	100.00
	65.29 (col. %)	63.54	59.57	52.38	63.52
Male	126	245	19	20	410
	30.73	59.76	4.63	4.88	100.00
	34.71	36.46	40.43	47.62	36.48
Total	363	672	47	42	1124
	32.30	59.79	4.18	3.74	
	100.00	100.00	100.00	100.00	

### 2.4. Sample weighting

The incorporation of a sample weighting technique into the modelling procedure in this study was done in order to ensure that the various bootstrap samples created were representative to that of the UWC student population. Since the results of this study could be used by the HIV Unit at UWC to further develop the HIV prevention programme at UWC, the results obtained in this study needed to be representative of the UWC student body.

A weight in statistics refers to the representation that a unit or observation carries. Weighting is usually used in the case where a specific unit or units within a sample (taken from some population) carry more influence on the results than other units. The sampling weights themselves also help with the bias carried over from the sampling procedure (Kirchoff, 2010).

As reported by Maletta, when scale-weighting is applied (as opposed to proportional-weighting), the estimates obtained from the sample were biased and did not represent the true population. This is also the case where the sample itself is a random sample as there could be errors associated with the sampling method and/or underlying bias (Kirchoff, 2010).

Luus et al. (2012) reported a significantly reduced bias and stable Mean Square Error (MSE) when weighting was applied to the sample. In their study they used a method of proportional-weighting in their analysis to account for non-respondents ‘to ensure that the weights sum to the correct population total’ (Luus et al., 2012, p. 86). In this study the proportional-weighting technique was applied and the formula for proportional-weighting is shown in Equation (3) :

(3) $$w_{i}=\;\frac{N_{i}/N}{n_{i}/n},\;$$

In Equation (3) the *i* represents a specific unit under study within the population (i.e. a subgroup of the population) and N would be the population total and *n* the sample total, therefore $N_{i}$ would be the unit total within the population and $n_{i}$ the unit total within the sample

The sampling weights used in this study were calculated based on the gender versus racial groupings, as shown for the training data set in Table 2. Since a total of 1700 bootstrap samples were created (i.e. 200 + 500 + 1000), only the sampling weights for the training data set are shown.

**Table 2.** Sampling weights for the training data set.

	Race			
	African/Black	Coloured	White	Indian/Asian
Female	21.09 (Sample %)	37.98	2.49	1.96
	24.11 (UWC %)	32.10	1.65	2.91
	1.14 (Weight)	0.85	0.66	1.49
Male	11.21	21.80	1.69	1.78
	17.84	17.90	1.12	2.38
	1.59	0.82	0.66	1.34

The sampling weights were obtained by dividing the UWC percentage by that of the sample percentage for each gender/race category. The UWC percentage remained constant in this calculation whereas the sample percentage changed based on the bootstrap sample. Each bootstrap sample contained unique gender and racial grouping sampling weights.

The variables gender and race, were the only variables that were used in the weighting procedure in this study. Previous research found that sexual activity prior to entering university was different for each gender group as well as the racial (population) groups (Blignaut, Vergnani and and Jacobs, 2014) and it was therefore decided to weight the training data to represent the gender and racial group proportions of the first-year entering students in 2010. (e.g. 24.11% of the entering students were black females in 2010 but in the sample this percentage was 21.09%).

Since this study made use of the binary logistic regression model only two possible outcomes were defined. The two outcomes or response variables were defined as whether a student was sexually active or not sexually active when entering university. In the logistic regression procedure modelled in this study, the outcome variable was assigned as a student not been sexually active. Therefore the predictor variables identified as significant in the logistic regression modelling procedure would be those predictor variables associated with a student who was not sexually active prior to entering university.

For the training data set 52% of students were found to be sexually active compared to 48% that were found to not be sexually active (see Table 3 and Table 4) when entering university. A breakdown of the sexually activity of students according to gender is given in Table 4.

**Table 3.** Sexual activity versus race in the training data set (unweighted data).

	Race				
	African/Black	Coloured	White	Indian/Asian	Total
Sexually Active	209 (count)	297	29	6	542
	38.56 (row %)	54.80	5.35	1.29	100.00
	61.83 (col. %)	47.44	65.91	19.44	51.92
Not Sexually Active	129	329	15	29	502
	25.70	65.54	2.99	5.78	100.00
	38.17	52.56	34.09	80.56	48.08
Total	338	626	44	36	1044
	32.38	59.96	4.21	3.45	
	100.00	100.00	100.00	100.00

**Table 4.** Sexual activity versus gender in the training data set (unweighted data).

	Gender		
	Female	Male	Total
Sexually Active	308 (count)	234	542
	56.83 (row %)	43.17	100.00
	45.77 (col. %)	63.07	51.92
Not Sexually Active	365	137	502
	72.71	27.29	100.00
	54.23	36.93	48.08
Total	673	371	1044
	64.46	35.54	
	100.00	100.00	

A total of fourteen predictor variables were included in the logistic regression modelling procedure and these are given in Table 5. All predictor variables had only two possible categorical responses.

**Table 5.** List of predictor variables and their responses.

Predictor Variable	Variable Code Name	Response/Categories	
Gender of student	gender	Male	Female
Do you personally know anyone with HIV/AIDS?	know_anyone_HIV	Yes	No
Do you feel that you know enough about HIV/AIDS?	know_enough_HIV	Yes	No
Have you ever taken an HIV test?	Taken_HIV	Yes	No
Do you intend to go for an HIV test?	intention_HIV_test	Yes	No
Accommodation during studies	Res	Stays in hostel	Does not stay in hostel
Matriculation province	Prov	Western Cape	All other Provinces
Use any drug in the last 30 days	drug_use	Yes	No
Age Group	age_group	16–19	20–24
Racial Group	racial_gr2	African	Not African
Do you use alcohol?	alcohol_use	Yes	No
Do you smoke?	smoke	Yes	No
Depressed more than 2 weeks in row	depressed	Yes	No
Importance of religion in influencing sexual activity	religion_vi	Very Important	Not so Important

For the variable racial group in Table 5, students were assigned as African and not African. This was done to ensure that the races of those students (i.e. white and Indian) whose overall sample size was too small to have an effect on the model predictor variables but would still be included as an initial predictor variable.

### 2.5. Variable selection methods

The variable selection methods used in this study are the forward, backward, stepwise, Newton–Raphson and Fisher variable selection methods. In each of the variable selection methods predictor variables are either dropped or added to the logistic regression based on a predefined significance level or alpha value.

The significance level for inclusion or exclusion of predictor variables was set at 0.01, therefore if the *p*-value of any predictor variable was below 0.01 then it was included in the logistic regression model. As suggested by Raftery, using a smaller significance level (i.e. 0.01) will ensure a model that has a more accurate predictive capability (Raftery, 1995).

### 2.6. Goodness-of-fit statistics

In order to assess the fit of the logistic regression model or models produced the AIC and BIC/SC goodness-of-fit statistics were used. The only difference between these two statistics (as used in SAS®) is the penalty term associated with each statistic (SAS, 2008):

(4) AIC=−2.ln(L)+2p,

(5) BIC/SC=−2.ln(L)+p.ln(n).

Both statistics incorporate the ‘deviance’ or log-likelihood function−2.ln(L) (Hilbe, 2009), which is a function that incorporates both the weight and frequency of each observation. For both Equations (4 and 5) the *p* value is assigned based on the number of parameters in the model and *n* is the number of observations.

When comparing either the AIC or BIC/SC goodness-of-fit statistics with those from other models, the model that produces the smallest AIC or BIC/SC goodness-of-fit statistic is the preferred model. It is worth noting that logistic regression models selected by using the AIC goodness-of-fit statistic will contain more predictor variables than the models selected using the BIC/SC goodness-of-fit statistic.

## 3. Results

### 3.1. Predictor variable inclusion in logistic models

Average inclusion of the predictor variables for all the bootstrap samples data sets is given in Table 6. Since the Newton–Raphson and Fisher variable selection methods did not incorporate an automated process of adding or dropping predictor variables from the model, all predictor variables were included in the logistic regression models for these two variable selection methods (i.e. high inclusion reported) and therefore their results is omitted from Table 6 (W = Weighted, UW = Unweighted).

**Table 6.** Average inclusion (given in %) of predictor variables in logistic regression models.

Variable	200 Bootstrap		500 Bootstrap		1000 Bootstrap		Total Average Inclusion	
	W	UW	W	UW	W	UW	W	UW
age_group	76.33	80.83	71.87	77.73	74.10	80.70	74.10	79.75
alcohol_use	99.33	98.17	97.20	95.60	98.67	97.20	98.40	96.99
depressed	9.00	11.00	8.07	11.60	9.67	12.53	8.91	11.71
drug_use	13.33	16.17	16.67	22.60	16.03	20.40	15.34	19.72
know_enough_HIV	5.50	3.00	2.40	1.47	3.60	1.53	3.83	2.00
taken_HIV_test	44.00	46.33	48.60	52.67	43.57	47.83	45.39	48.94
intention_HIV_test	1.50	0.83	1.53	1.13	2.23	1.57	1.75	1.18
know_anyone_HIV	15.17	18.00	15.80	15.53	12.43	12.23	14.47	15.25
racial_gr2	63.50	54.17	62.40	51.00	62.87	52.53	62.92	52.57
religion_vi	100.00	100.00	98.80	100.00	99.70	99.90	99.50	99.97
smoke	41.33	42.67	41.13	44.73	41.37	44.73	41.28	44.04
gender	19.00	16.17	21.07	18.47	20.90	16.40	20.32	17.01
prov	11.00	5.83	10.40	8.67	11.33	8.83	10.91	7.78
res	4.00	2.50	0.93	1.13	1.67	1.97	2.20	1.87

The average inclusion percentage for each predictor variable in Table 6 was calculated by averaging the inclusion of each of the variable selection methods across the various bootstrap sample models produced. The predictor variables: age group of the student, consumption of alcohol and the importance of religion in influencing their sexual behaviour all reported the highest inclusion across all the bootstrap sample models.

The total average inclusion for these three predictor variables across the 200, 500 and 1000 bootstrap samples data sets was 76.93%, 97.69% and 99.73% respectively. As an example, of the 17000 logistic regression models produced (i.e. 10 models constructed per sample), the age group of the student was found to be significant in 76.93% of these logistic regression models (approximately 13078 models).

Slight differences were observed for the inclusion of predictor variables between weighted and unweighted data as shown in Table 6. The predictor variable racial_gr2 produced the highest inclusion difference (average of 10.35%) between the models produced from the weighted and unweighted data (i.e. higher inclusion reported for the weighted data).

Only six variables (age_group, alcohol_use, taken_HIV_test, racial_gr2, religion_vi and smoke) reported average inclusion above 40% for the logistic regression models produced from the bootstrap samples. Further inspection of the probabilities produced for these six predictor variables showed that all their probability values were less than the 0.01 significance level used. Therefore the final logistic regression model is based on these six predictor variables.

Seven significant predictor variables were identified using only the original data set (i.e. no bootstrapping, using the three variable selection methods forward, backward and stepwise, with and without sample weighting). These seven predictor variables were: age_group, alcohol_use, taken_HIV_test, racial_gr2, religion_vi, smoke and drug_use. Whether a student had used drugs in the 30 days prior to completing the questionnaire was identified in the model for the training data set and not in those models produced from the bootstrapped samples.

**Figure 1.**
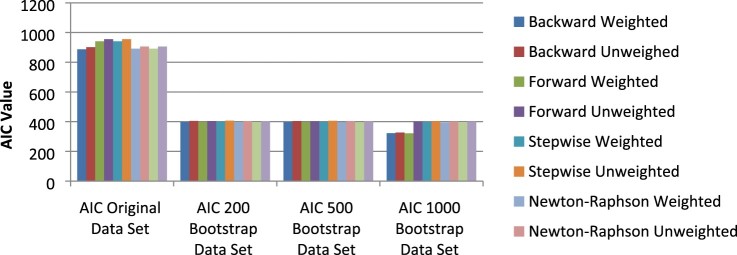
AIC values across data sets.

**Figure 2.**
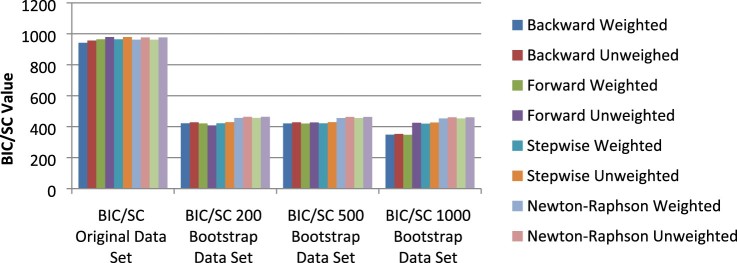
BIC/SC values across data sets.

In order to compare the models produced from the bootstrap samples, the AIC and BIC/SC values of the logistic regression model constructed on the original data set were also included in the analysis. The values for AIC and BIC/SC produced for the various logistic regression models is given in Figures 1 and 2. As can be seen in Figures 1 and 2, a substantial decrease in the values for AIC and BIC/SC between the original data set and the bootstrap samples was reported.

Bootstrapping produced significantly smaller values for AIC and BIC/SC, almost half the value of that of the original data set. The values for AIC and BIC/SC remained somewhat constant over the bootstrap samples; no major increase or decrease was noticed for these values across the bootstrap sample sizes except for the backward weighted, unweighted and forward weighted values for the 1000 bootstrap sample.

Although the 1000 bootstrap sample did provide smaller fit statistic values for the backward weighted and unweighted and forward weighted logistic regression models, the predicted probabilities produced for these models based on the data of the validation sample data set were found to result in inaccurate classifications. Low classification percentages were reported for these logistic regression models for both sexually active and not sexually active students. These low classification percentages led to the conclusion that these logistic regression models were unstable in their predictions.

**Figure 3.**
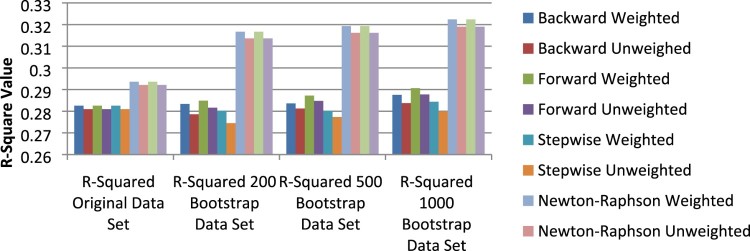
$\;\;R^{2\;}$values across data sets.

The coefficient of determination ($\;\;R^{2\;}$) also provided a good indication of which of the logistic regression models produced improved model estimates; this is shown in Figure 3. The most notable change in the value of $\;\;R^{2\;}$ was that of the Newton–Raphson and Fisher models, once bootstrapped data sets were introduced; Figure 3 shows a noteworthy difference in the value for these methods compared to all other variable selection methods.

The $\;\;R^{2\;}$ value marginally increased as the bootstrap sample size increased and more notably, all weighted variable selection methods produced slightly larger $\;\;R^{2\;}$ values than that of the associated unweighted variable selection method regardless of bootstrap sample size or whether bootstrapping even occurred. This larger $\;\;R^{2\;}$ value associated with weighted variable selection methods pointed to the efficacy of sample weighting as a means of providing a logistic regression model that better fits the data.

With the exception of the three models of the 1000 bootstrap sample previously mentioned, no notable difference was observed for the goodness-of-fit statistic values of the bootstrap samples. The estimate values of the predictor variables, as well as their probability values, were all similar across the bootstrap samples, therefore leading to the conclusion that a bootstrap sample size greater than 200 did not provide a greater accuracy than that of a 200 bootstrap sample size.

Therefore, taking all the previous conclusions reached in this study into consideration, the logistic regression model (further referred to as the stable logistic regression model) put forward is that of the weighted 200 bootstrap sample data set based on the forward, backward and stepwise variable selection methods, with the predictor variables: age _group; alcohol_use; taken_HIV_test; racial_gr2; religion_vi; and smoke. The parameter coefficients and standard errors of these parameters are given in Table 7.

**Table 7.** Stable Logistic Regression model estimates.

Parameter	Coefficient	Standard Error
Intercept	−1.220083	9.507995
age_group	0.720440	0.201313
alcohol_use	0.698189	0.135473
taken_HIV_test	−0.466004	0.135816
racial_gr2	−0.561591	0.140210
religion_vi	0.701504	0.132257
smoke	0.568500	0.166486

Since including sample weighting proved to be more effective, estimates for the six predictor variables were obtained from the weighted logistic regression models produced from the forward, backward and stepwise variable selection methods for the 200 bootstrap samples.

### 3.2. Final logistic regression model

A comparison was carried out in order to compare accuracy of the stable logistic regression model put forward (i.e. based on the six identified predictor variables). This was done by constructing and comparing a logistic regression model based on the original data set with that of the stable logistic regression model. Table 8 provides the classification percentages of these two logistic regression models.

**Table 8.** Correct classification percentages.

	Sexually Active	Not Sexually Active
Stable Logistic Regression Model	75.41%	63.41%
Original Data Set Logistic Regression Model	81.97%	21.95%

For the stable logistic regression model, 75.41% of sexually active students were correctly classified and 63.41% of not sexually active students were correctly classified. For the logistic regression model constructed from the original data set, 81.97% of sexually active students were correctly classified and 21.95% of not sexually active students were correctly classified (see Table 8).

A slightly higher classification percentage was noted for the original data set model compared to that of the stable model for sexually active students, indicating that the original data set model was slightly more accurate at classifying sexually active students. A sizeable difference, was however, observed between the two models for the classification of not sexually active students (i.e. the outcome modelled in this study). The original data set logistic regression model correctly classified only 21.95% students compared to 63.41% for the stable logistic regression model.

This notable difference in the classifications of the original data set logistic regression model indicates that this logistic regression model is somewhat unstable when classifying students according to their sexual activity. The stable logistic regression model, however, produced more reliable classification estimates, indicating that this logistic regression model is the more stable and more accurate logistic regression model of the two.

## 4. Conclusion

The logistic regression modelling procedure carried out in this study included a bootstrapping method, a sample weighting process and predictor variable selection methods (forward, backward, stepwise, Newton–Raphson and Fisher).

Smaller values were observed for the goodness-of-fit statistics; Akaike’s Information Criterion (AIC) and Bayesian Information Criterion (BIC/SC) for the logistic regression models produced from the bootstrapped data set samples. The bootstrap models produced fit statistic values that were less than half that of the original data set models (smaller AIC and BIC/SC values preferred) (see Figures 2 and 3). This showed that models produced from bootstrapped data fitted the data better than models produced without bootstrapping.

Weighted logistic regression models produced from the bootstrapped data also further produced AIC and BIC/SC values that were smaller than those of unweighted models produced from bootstrapped data. The $\;R^{2}\;$ value was also examined in order to back up the conclusion reached from the AIC and BIC/SC goodness-of-fit statistics.

No considerable increase in the value for $\;R^{2}$ was reported between the original data set model and the bootstrap sample data models. Although these increased values were not notable enough to definitively conclude better fitting models based on$\;R^{2}$, it was observed that bootstrapped data did produce larger $\;R^{2}$ values, however small these values were.

Larger $\;R^{2}\;$ values were also reported for weighted logistic regression models than unweighted logistic regression models in a comparison of the models produced from bootstrapped data. This further emphasised the point that incorporating a sample weighting process and a bootstrapping procedure in a logistic regression modelling procedure will produce models that will better fit the data, hence increasing the predictive capability of the logistic regression models.

For the stable logistic regression model, the inclusion of each of the predictor variables across the bootstrap samples was analysed since it had been determined that bootstrapping produced more accurate logistic regression models. The six predictor variables age_group, alcohol_use, taken_HIV_test, racial_gr2, religion_vi and smoke were the most used predictor variables across all of the bootstrap samples (see Table 5). The Newton–Raphson and Fisher variable selection methods identified only two predictor variables in all of the models produced for these variable selection methods and were thus determined to be unreliable.

The size of the bootstrap sample was also determined not to be more accurate for sample sizes greater than 200. The AIC, BIC/SC and $\;R^{2}$ values did not change considerably over the bootstrap sample sizes, remaining somewhat constant as the bootstrap sample size increased. Therefore, bootstrap sample sizes greater than 200 were determined not to provide logistic regression models that better fitted or provided model estimates that were significantly different to a bootstrap sample size of 200.
